# The Effect of Training Status on Adaptations to 11 Weeks of Block Periodization Training

**DOI:** 10.3390/sports8110145

**Published:** 2020-10-31

**Authors:** Alexander B. Wetmore, Paul A. Moquin, Kevin M. Carroll, Andrew C. Fry, W. Guy Hornsby, Michael H. Stone

**Affiliations:** 1 Center of Excellence for Sport Science and Coach Education, Department of Sport, Exercise, Recreation and Kinesiology, East Tennessee State University, Johnson City, TN 37614, USA; moquin@etsu.edu (P.A.M.); carrollk@etsu.edu (K.M.C.); stonem@etsu.edu (M.H.S.); 2 Osness Human Performance Laboratories, Department of Health, Sport, and Exercise Sciences, University of Kansas, Lawrence, KS 66045, USA; acfry@ku.edu; 3 College of Physical Activity and Sport Sciences, West Virginia University, Morgantown, WV 26505, USA; William.hornsby@mail.wvu.edu

**Keywords:** strength, relative strength, resistance training

## Abstract

Some controversy exists as to the most efficacious method of training to achieve enhanced levels of sport performance. Controversy concerning the efficacy of periodization and especially block periodization (BP) likely stems from the use of poorly or untrained subjects versus trained who may differ in their responses to a stimulus. The purpose of this study was to investigate the effect of training status on performance outcomes resulting from 11 weeks of BP training. Fifteen males were recruited for this study and placed into strong (age = 24.3 ± 1.9 years., body mass (BM) = 87.7 ± 8.7 kg, squat: body mass = 1.96 ± 0.16), moderate (age = 25.3 ± 2.7 years., body mass = 100.2 ± 15.5 kg, squat: body mass = 1.46 ± 0.14), or weak (age = 23.2 ± 3.9 yrs., body mass = 83.5 ± 17.1 kg, squat: body mass = 1.17 ± 0.07) groups based on relative strength. Testing was completed at baseline, and after each block which consisted of 1 repetition maximum (1RM) squat, 0 kg static jump (SJ), 0 kg countermovement jump (CMJ), 20 kg SJ, and 20 kg CMJ. Absolute and relative strength were strongly correlated with rates of improvement for absolute strength, relative strength, 0 kg, and 20 kg vertical jumps. All subjects substantially improved back squat (*p* < 0.001), relative back squat (*p* < 0.001) with large–very large effect sizes between groups for percent change favoring the weak group over the moderate and strong group for all performance variables. All subjects showed statistically significant improvements in 0 kg SJ (*p* < 0.001), 0 kg CMJ (*p* < 0.001), 20 kg SJ (*p* = 0.002), and 20 kg CMJ (*p* < 0.001). Statistically significant between group differences were noted for both 20 kg SJ (*p* = 0.01) and 20 kg CMJ (*p* = 0.043) with the strong group statistically greater jump heights than the weak group. The results of this study indicate BP training is effective in improving strength and explosive ability. Additionally, training status may substantially alter the response to a resistance training program.

## 1. Introduction

Controversy exists as to the most efficacious method of training to achieve enhanced levels of sport performance characteristics, especially as it pertains to strength and power [1,2,3]. The majority of reviews of the literature [4,5,6,7,8,9,10,11] including several meta-analyses [10,11] have consistently concluded that a “periodized” training concept offers advantages over non-periodized processes.

However, some controversy concerning the periodization models exists [11]. There are only two models of periodization, Traditional (Classic) and Block [4,12]. Traditional periodization allows for simultaneous alterations in a variety of fitness characteristics, whereas single factor block periodization takes a more consecutive approach in which one or a few compatible characteristics are developed before emphasizing a different set of characteristics [5,6,12].

Much of this controversy stems from confusion of periodization with programming [4]. It should be noted that periodization is a conceptual paradigm that deals with (1) Fitness phases and (2) Time lines for implementation of the fitness phases. There are two basic (general) premises of the periodization concept: (1) less specific to more specific and (2) higher volume to lower volume [5,6,13]. Based on past [14,15], and particularly recent evidence [13,16,17], it is becoming increasingly clear that Block Periodization provides superior results when properly programmed.

Briefly Simple Block periodization consist of three primary phases, Accumulation (general preparation), Transmutation (special preparation), and Realization (competition and taper). Periodization is supported mechanistically by several basic hypotheses/theories of describing an organism’s reaction to a specific stimulus [4,12]. These conceptual mechanisms include stimulus-fatigue-recovery adaptation, the general adaptation syndrome (GAS) and specifically for strength power training development of hypertrophy, then basic strength then power [4,12,18,19].

Much of the controversy concerning the efficacy of periodization and especially block periodization likely stems from the use of trained versus poorly or untrained subjects and the use of programming techniques used to drive the periodization model [4]. For example: recently Painter et al. [16,17], and particularly Carroll et al. [13], have provided evidence that training to failure using RM zones may inhibit gains in maximum strength, rate of force development (RFD) and power. Compared to non-failure, training to failure can produce a relatively high degree of training monotony and strain that is reflected to greater extent in negative physiological/metabolic responses (e.g., testosterone, cortisol, neutrophil: leucocyte ratios, etc.). This negative aspect of adaptation noted with training to failure is also in agreement with recent studies indicating an extended recovery necessary for training to failure [20,21,22]. Extended recovery may inhibit adaptation or potentiate non-functional overreaching or overtraining [13,17], particularly when applied to a sport environment with other training in addition to the resistance program.

Training level may be a major influence on training outcomes as (1) untrained subjects using the same stimulus tend to gain strength at a faster rate than trained [10,23]; (2) previous training producing increased maximum strength may potentiate further gains in power when power training is emphasized [24,25,26]; (3) training combinations using a strength plus power emphasis can potentiate both strength and power gains [4,12], and (4) initial gains in hypertrophy may be due to changes in edema and swelling [27]. Thus, the purpose of this study is to study the effect of training status on adaptation to block periodization resistance training.

## 2. Materials and Methods

### 2.1. Subjects

Based on the results of previous investigations [13], power analysis for repeated measures ANOVA with a moderate effect size was calculated (α = 0.05, f = 0.9, number of groups = 3, number of measurements = 5). It was determined that a sample size of 12 was needed (Gpower vers. 3.0.10). Fifteen healthy males of various training experience volunteered for this study. Correlations indicate a strong and consistent negative relationship between the initial 1RM and gains in performance. This would indicate that weaker subject’s progress at a greater rate than stronger subjects. Considering these correlations subjects were divided into three groups based on their initial 1RM squat. Based on the criteria outlined by Suchomel et al. 2018, subjects were grouped according to their relative (1RM/Body mass) squat [12]. Subjects (*n* = 7) unable to back squat at least 1.25 kg/kg were considered weak (age = 23.2 ± 3.9 yrs., BM = 83.5 ± 17.1 kg, squat: BM = 1.17 ± 0.07). A 1 RM back squat between 1.25–1.75kg/kg were considered moderate (*n* = 4) (age = 25.3 ± 2.7 years, BM = 100.2 ± 15.5 kg, squat: BM = 1.46 ± 0.14). A 1 RM back squat greater than 1.75 kg/kg were considered strong (*n* = 4) (age = 24.3 ± 1.9 years, BM = 87.7 ± 8.7 kg, squat: BM = 1.96 ± 0.16). This study was approved by the university Institutional Review Board (IRB) and all subjects were informed of the benefits and risks of the investigation prior to signing an institutionally approved informed consent document to participate in the study.

### 2.2. Procedures

A block periodization design was used for the resistance training program as it has been previously shown to be effective in developing maximum strength and power [14,15,16,17]. All subjects completed one baseline testing session, and a testing session following each of the four training blocks. Subjects with no prior training history underwent a two-week familiarization period in which they learned each of the exercise techniques prior to beginning training to account for learning effects. Pre-testing took place one week before the beginning of the training intervention. Each post-block testing session was completed on the last training session of the block with two days separating post-block testing and the beginning of the next block of training. All testing sessions were completed at the same time of day and in the same order of tests. Training loads were consistently tracked. A mixed within and between subject design was selected to examine the effect of the training program on performance characteristics both within subjects and between groups.

All groups completed the same non-failure strength training program and testing scheme. The training program followed a single factor block periodization model and was programmed with an emphasis on strength and power development.

The subjects completed three resistance sessions per week (Monday, Wednesday, and Friday) and two sprint sessions per week (Tuesday and Thursday). The sprint warm-up and program was designed to simulate a sport practice environment.

The training program contained three sequential summated microcycles or blocks (strength-endurance, maximal strength, and power) including a functional overreach and taper. Additionally, each summated microcycle contained heavy and light days to both manage fatigue and ensure a spectrum of power outputs. Lastly, all training loads were selected using relative intensities (% set-rep best). The training program is shown in Table 1.

The exercise selection for both groups is shown in Table 2.

Pre-intervention testing was conducted one week prior to the start of the intervention and concluded 48 h prior to the start of the intervention for the participant. Pre-testing included hydration status, jump height and dynamic strength. Hydration was tested using a refractometer (Atago, Tokyo, Japan). Dehydration has been shown to have a negative effect on performance, cognitive abilities and ultimately testing results [28]. Subjects were required to have a USG > 1.20 to begin testing.

Static jumps (SJ) and counter movement jumps (CMJ) were assessed using dual force plates (2 cm × 91 cm × 45.5 cm) sampling at 1000 Hz (Rice Lake Weighing Systems, Rice Lake, WI, USA). Both unweighted (Polyvinyl chloridepipe) and weighted (20 kg barbell) jumps were collected for both SJ and CMJ. The PVC pipe and barbell were used to eliminate arm swing and to standardize testing conditions between subjects [13]. Subjects performed a SJ from an internal knee angle of approximately 90° [13]. SJ testing followed a standardized warm-up [29] and subjects performed two warm-up jumps for the unweighted SJ at 50% and 75% effort. Subjects then performed at least two SJ at 100% effort. If jump heights differed by greater than 2 cm, then additional trials were performed until two unweighted jumps within 2 cm of each other. Once complete, subjects began testing weighted jumps using the same procedure as unweighted jumps. Unweighted and weighted CMJ were performed using the same procedure as SJ.

Dynamic strength was assessed via 1 repetition maximum back squat. Prior to the first attempt, a standardized warm-up was performed [30]. This standardized warm up is shown in Table 3 below.

The testing percentages were based on self-estimated 1 RM and the trial and error method for 1 RM [31]. After a successful attempt, subjects continued to attempt progressively heavier loads until a true 1 RM was reached. Attempts were deemed successful if the line from the top of the knee to the hip crease was parallel (or below) with the floor. Squat depth was determined by two experienced certified strength and conditioning specialists.

Post block testing consisted of hydration, performance testing and dynamic strength measures. Post block testing was completed after the 3-week strength endurance (SE) block, 4-week maximum strength (MS) block, the 1-week functional overreach (FOR) and the 3-week taper. Post block testing was completed on the last scheduled training session of every block (Friday). Testing consisted of performance (jumps), and dynamic strength (1 RM squat). After testing, subjects completed the remainder of their scheduled day 3 training session (with the exception of back squat).

### 2.3. Statistics

A series of 3 × 5 two-way mixed design ANOVAs (group × time) was used for this study with an alpha level of *p <* 0.05. In addition to null hypothesis testing, magnitudes of effect were calculated using Cohen’s *d* effect sizes. Additionally, correlational statistics were calculated using Pearson’s *r* to assess the relationships between training status and performance. All statistics were calculated using JASP (JASP version 0.11.1.0, manufacturer, city, country). Cohen’s *d* magnitude thresholds and correlation thresholds are shown in Table 4. [32].

Intraclass correlations for all force plate measures were considered excellent (0.986–0.994) [32]. Reliability statistics for our lab are shown in Table 5.

## 3. Results

### 3.1. Correlation between Strength Levels and Adaptations to Training

All dependent variables met the assumptions of normality and sphericity at the level of significance. When investigating the relative rates of change in performance between groups, several distinctions can be made. In dynamic strength, for example, both the moderate and weak groups showed their greatest change from baseline to the end of the SE phase (Table 6). However, the strong group showed its greatest improvement from the end of SE to the end of the MS phase. Similar trends were found for relative dynamic strength but with the strong group’s greatest improvement from the end of the FOR to the taper phase. Taken together with all performance variables, there is a clear difference in rates and timing of adaptation to a stimulus between strength groups.

Statistically significant correlations were found between both absolute and relative strength levels and various rates of physical improvements. For example, initial absolute squat strength was strongly correlated with absolute squat pre/post change (*r* = −0.738), relative squat pre/post change (*r* = −0.767) pre/post change for both 0 kg (*r* = −0.555) and 20 kg SJ (*r* = −0.608), and peak power pre/post change for both 0 kg (*r* = −0.709) and 20 kg (*r* = −0.709) SJ. Additionally, strong correlations were noted between absolute strength and early (first block) strength changes (*r* = −0.524) as well as post-taper improvements in SJ (*r* = −0.526) and CMJ (*r* = −0.517). Similar relationships exist between relative strength levels and physical adaptations to training including, pre/post change in absolute strength (*r* = −0.751), pre/post change in relative strength (*r* = −0.727), pre/post change in 20 kg CMJ (*r* = −0.526), pre/post change in 0 kg SJ peak power (*r* = −0.586) and 20 kg SJ peak power (*r* = −0.589), and early (first block) changes in absolute (*r* = −0.544) and relative strength (*r* = −0.517). A full list of statistically significant correlations is shown in Table 7.

### 3.2. Vertical Jump Testing

Both weighted and unweighted vertical jumps were measured before and after each block of training. The 0 kg SJ showed a statistically significant main effect for time increase in jump height (*p <* 0.001). While there were not statistically significant differences between groups for 0 kg, the strong group improving 7.6%, the moderate group improving only 0.3%, and the weak group improving 25.6% over the course of the study. It is worth noting that two subjects in the moderate group improved (4.8 cm and 2.3 cm, respectively) while two decreased (−3.9 cm and −2.9 cm, respectively) likely confounded the overall group mean. Similar results were found for the 0 kg CMJ with a significant effect for all subjects over the course of the study (*p <* 0.001) and non-significant differences between groups. However, noticeable differences in the percent changes were noted with the strong group improving 9.9%, moderate group improving 11.0%, and the weak group improving 23.8% over the course of the study.

The 20 kg static jumps showed statistically significant improvements for all subjects (*p* = 0.002) and statistically significant effects of strength level on jump height from baseline to post-testing (*p* = 0.01). The strong group improved 4.8%, the moderate group improved 8.4%, and the weak group improved 28.2%. Similarly, the 20 kg CMJ showed statistically significant improvements for all subjects (*p <* 0.001) and statistically significant differences between groups (*p* = 0.043). The strong group improved 9.6%, the moderate group improved 9.0%, and the weak group improved 27.9% over the course of the study from pretesting to post testing.

Full results for all vertical jumps are shown in the Table 8 and Table 9 below.

### 3.3. Peak Power

Peak power (PP) was measured across all four vertical jump conditions. PP statistically improved for 0 kg SJ, 20 kg SJ, 0 kg CMJ, and 20 kg CMJ (*p <* 0.001). Percent change in PP for each jump condition is listed in the Table 10 below.

### 3.4. Dynamic Strength

**Figure 1:**
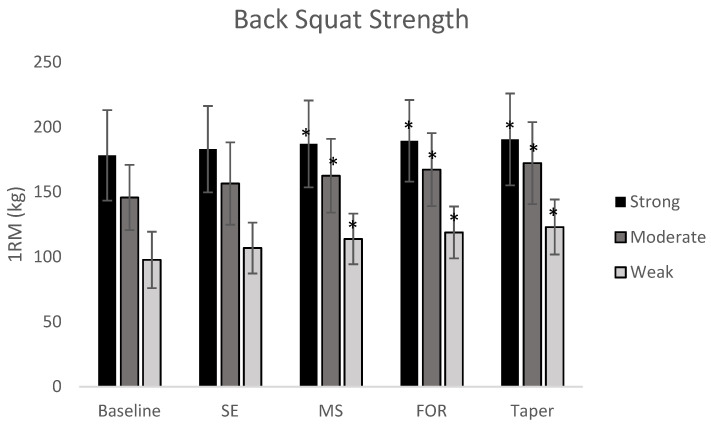
Block to Block changes in absolute back squat. * indicates a significant improvement from the baseline strength values (*p* < 0.05).

**Figure 2:**
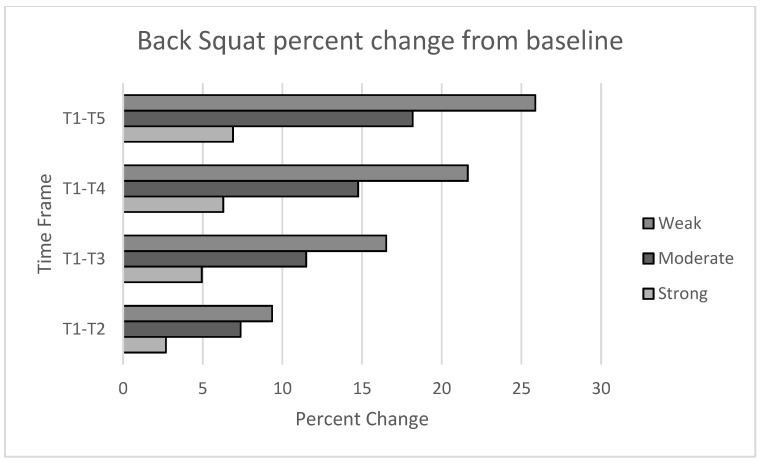
Block to block percent change in absolute back squat.

Dynamic strength showed significant improvements for all subjects (*p* = 0.002). Post hoc analysis showed significant differences between groups with small to large effects. The largest improvement was noted in the weak group (25.9%) with smaller improvements in the moderate (18.2%) and strong groups (11.3%). All results are shown in the Table 11 and Table 12. Changes in maximum strength are depicted in Figure 1 and Figure 2

## 4. Discussion

The vast majority of existing reviews of the literature [5,6,7,8,9,10,11] and several meta-analyses [10,11,33], have consistently concluded that a “periodized” training concept offers advantages over non-periodized processes. The results of our current study support the previous literature in the effectiveness of a periodized program in enhancing maximum strength and power. The sequential programming approach used within BP has also been termed phase-potentiation [15]. Phase potentiation programming is a process by which programming alterations in a concentrated load in one block may further potentiate the adaptations in the subsequent blocks due to accumulated residual training effects [5,6] Power, along with impulse, has previously been defined as a most important attribute for athletic performance [15]. As noted in the introduction, development of maximal strength may potentiate further gains in power. Our results support this theory as subjects realized early gains in strength after the SE and MS blocks which led to large improvements in jump height and PP during the taper. It should be noted however, the strong group generally realized the greatest gains as a result of the taper (Table 6). These results, particularly for the strong group, are indicative of the shift in emphasis over a BP program from general strength endurance towards realizing maximum strength and power in the later phases of training, along with a volume reduction. Similar results have been found previously by Carroll et al., (2018) who employed a very similar BP training program. In their results, subjects substantially improved their scaled PP from pre to post (*p* = 0.003) as well as during the final phase of the program (taper) (*p* = 0.026) [13]. Our current findings along with previous findings support the efficacy of a BP model for maximizing strength and power, especially in the later phases of training.

The results of this study highlight the importance of training status on adaptation to a training stimulus. Statistically significant correlations were found between initial strength levels (both absolute and relative) and improvement in strength (absolute and relative) over time. Specifically, strong negative correlations were found between initial strength levels and percent change in maximum strength and vertical jump ability indicating that weaker individuals improve at a greater rate than stronger individuals. These results are supported by those of Ahtiainen et al. (2003) who compared strength athletes and non-athletes over the course of 21 weeks of training. The results noted a 20.9% increase in maximum strength for non-athletes and only 3.9% in the strength athlete group [23]. Additionally, a meta-analysis by Rhea et al. (2003) notes different responses to training based on training status [10]. Specifically, previously trained subjects require higher intensities for maximal gains compared to their untrained counterparts. However, one very interesting finding of the current study was the correlation between strength levels and both early and late phase development. Absolute strength was negatively correlated with early strength development (T1-T2) (r = −0.524) and negatively correlated with later improvements in jump height (T4-T5) (r = −0.526 for 0 kg SJ and r = −0.517 for 20 kg CMJ) showing greater gains for weaker subjects than stronger ones. Theoretically, these correlations support the proposed mechanisms of phase-potentiation as early gains in strength for untrained subjects manifests itself via power gains later in the program. Both the moderate and weak groups showed their greatest improvements in maximum strength after the SE block. However, the strong group showed its greatest change in maximum strength after the MS block indicating that stronger individuals may not realize substantial improvements in maximum strength until a more specific stimulus is applied. Lastly, there were marked differences in relative strength changes during the taper phase. While all groups showed improvement during the taper, only the strong group showed its greatest improvement in relative strength during the realization phase. A major goal of a taper is the reduction in volume which may dissipate fatigue as well as improve relative strength due to residual training effects. The gain in relative strength may contribute to increased power development which is fundamental for sport success during important competition periods. Previous research has proposed several mechanisms which may contribute to this observed increase in power during a taper. There is typically a reduction in fatigue accompanying volume reductions which may lead to increased performance in keeping with the fitness fatigue paradigm [34]. One possible mechanism which may also contribute to the increase in power development during a taper is a shift in myosin heavy chain (MHC) isoform. Several studies have cited a shift from slower to faster isoforms during periods of reduced training [35,36,37]. Andersen et al. (2000) studied changes in MHC after three months of heavy resistance training and again after three months of detraining [35]. The results showed a significant shift of type IIx MHC to MHC IIa after resistance training with significant hypertrophy of the type II fibers. Interestingly, after three months of detraining, MHC isoforms had shifted back towards IIx with values statistically higher than baseline. The observed fiber type distribution mirrored the changes in MHC isoform. The results of this study lend support to the possibility of a IIx “super compensation” after a period of reduced training and may partially explain the increase in power potential during a taper as IIx MHC are more explosive than type I or IIa. Additionally, residual training effects resulting in maximum strength may last well into a period of reduced training. The maintenance of maximum strength paired with a possible shift of fiber type towards more powerful MHC isoforms, provide a sound basis for including a taper during periods of time in which power is the goal, such as important competitions. However, given the results of our study, it is possible that in developing athletes, taper responses may differ, as we observed different changes in relative strength levels and PP between the strong and the moderate/weak groups.

Lastly, previous authors have proposed that greater levels of variation or advanced training tactics may be beneficial for more advanced athletes. For example, in their review, Kraemer and Ratamess (2004) state that advanced lifters progress at much slower rates compared to lesser trained individuals as they begin to approach their genetic ceiling [38]. The authors also note that small changes in strength may require large amounts of training time, but the time can be worth the effort because small changes may be the difference between winning and losing. Therefore, the authors state that advance training is more complex and requires greater variation specific to training goals.

One possible limitation of the current study is the limited sample size. To better understand the effect of training status on adaptations to training, further research with greater sample sizes is warranted. One additional limitation of the current study is the relatively short duration of 11 weeks (one stage). While the current study is one of the longest-term studies currently available, it would be very informative to continue to follow adaptations to a program multiple stages in length.

## 5. Conclusions

The findings of the current study demonstrate the effectiveness of a BP training program in improving both strength and power capabilities across different training levels. An important concept in power development is that increases in maximum strength before a realization phase emphasizing power will potentiate power adaptations [15,24,39]. Our results indicate a marked difference in rates of improvement between different training level groups agreeing with this concept. Specifically, initial strength levels were negatively correlated with rates of improvement in strength and power. Therefore, it is recommended that coaches and sport scientists use a periodized training program with their athletes. Additionally, we recommend practitioners implement a regular monitoring program to better understand potential adaptations to a resistance training program based on training status. Lastly, as athletes improve their training status and begin to approach their genetic potential, more advanced training tactics may be warranted to continue to promote adaptation to a specific stimulus.

## Figures and Tables

**Table 1:** Resistance training program, * signifies down set at 50% of target weight after major exercise (squats, bench, mid-thigh pull).

Training Block	Week	Sets × Reps	Day 1 and 2	Day 3
Strength-Endurance	1	3 × 10	80%	70%
	2	3 × 10	85%	75%
	3	3 × 10	90%	80%
Maximum Strength	4	3 × 5 (1 × 5) *	85%	70%
	5	3 × 5 (1 × 5) *	87.5%	72.5%
	6	3 × 5 (1 × 5) *	92.5%	75%
	7	3 × 5 (1 × 5) *	80%	65%
Overreach	8	5 × 5	85%	75%
Speed-Strength	9	3 × 3 (1 × 5) *	87.5%	67.5%
	10	3 × 2 (1 × 5) *	85%	65%
	11	2 × 2 (1 × 5) *	65% & 60%	-

**Table 2:** Resistance Training Exercise Selection.

Training Block	Day 1	Day 2	Day 3
Strength–Endurance	Back Squat, Overhead Press, Bench Press, DB Tricep Ext.	CG MTP (3 × 5), CG SLDL, BB Bent Over Row, DB Bent Lateral Raise	Back Squat, Overhead Press, Bench Press, DB Tricep Ext.
Maximum Strength	Back Squat, Push Press, Incline Bench Press, Wtd. Dips	CG MTP, Clean Pull, SG SLDL, Pull Ups	Back Squat, Push Press, Incline Bench Press, Wtd. Dips
Overreach	Back Squat, Push Press, DB Step Ups, Bench Press	CG CM Shrug, Clean Pull, CG SLDL, SA DB Bent Over Row	Back Squat, Push Press, DB Step Ups, Bench Press
Speed-Strength	Back Squat + Rocket Jumps, Push Press, Bench press + Medicine Ball Chest Pass (4.5 kg)	CG MTP, CG CM Shrug, Medicine Ball Countermovement Toss for height (4.5 kg)	Back Squat + Rocket Jumps, Push Press, Bench press + Medicine Ball Chest Pass (4.5 kg)

DB = dumbbell, CG = clean grip, MTP = mid-thigh pull, BB = barbell, Ext = extension, Wtd. = weighted, SG = snatch grip, SLDL = stiff-legged deadlift, SA = single arm, CM = counter-movement.

**Table 3:** Warm-up protocol prior to all 1 RM lift attempts and rest time after all warm-up sets.

5 × 30% of 1RM *	3 × 50% of 1RM *	2 × 70% of 1RM *	1 × 80% of 1RM *	1 × 90% of 1RM *
1 min	1 min	2 min	3 min	3 min

* 1 RM weight for untrained subjects will be based on the participant’s estimated 1 RM.

**Table 4:** Magnitude thresholds for Cohen’s *d* and Pearson’s *r.*

Cohen’s *d*	Effect	Pearson’s *r*	Relationship
0–0.2	Trivial	0.0–0.1	Trivial
0.2–0.6	Small	0.1–0.3	Small
0.6–1.2	Moderate	0.3–0.5	Moderate
1.2–2.0	Large	0.5–0.7	Large
>2.0	Very Large	0.7–0.9	Very Large
--	-	1.0	Perfect

**Table 5:** Reliability Statistics.

Variable	ICC
0 kg SJ JH	0.986
20 kg SJ JH	0.994
0 kg CMJ JH	0.991
20 kg CMJ JH	0.992
0 kg SJ Peak Power	0.982
20 kg SJ Peak Power	0.984
0 kg CMJ Peak Power	0.991
20 kg CMJ Peak Power	0.994

**Table 6:** Blocks with greatest percent changes.

Group	0 kg SJ	20 kg SJ	0 kg CMJ	20 kg CMJ	Absolute Back Squat	Relative Back Squat
Weak	Taper	SE	Taper	Taper	SE	SE
Moderate	SE	Taper	Taper	FOR	SE	SE
Strong	Taper	Taper	Taper	SE	MS	Taper

**Table 7:** Correlations between initial strength levels and training adaptations.

Variable	Variable	Pearson’s *r*	*p*-Value
Pre-Absolute 1 RM	Pre-Relative Squat	−0.833	<0.001
Pre-Absolute 1 RM	Absolute Squat Pre/Post % change	−0.738	0.002
Pre-Absolute 1 RM	Relative Squat Pre/Post % change	−0.767	<0.001
Pre-Absolute 1 RM	0 kg SJ pre/post % change	−0.555	0.0032
Pre-Absolute 1 RM	20 kg SJ pre/post % change	−0.608	0.016
Pre-Absolute 1 RM	T1-T2 Abs. Squat % change	−0.524	0.045
Pre-Absolute 1 RM	0 kg SJ T4-T5 % change	−0.526	0.044
Pre-Absolute 1 RM	20 kg CMJ T4-T5 % change	−0.517	0.049
Pre-absolute 1 RM	0 kg SJ Peak Power pre/post % change	−0.709	0.003
Pre-Absolute 1 RM	20 kg SJ Peak Power pre/post % change	−0.709	0.003
Pre- Relative 1 RM	Absolute 1RM pre/post % change	−0.751	0.01
Pre-Relative 1 RM	Relative 1RM pre/post % change	−0.727	0.002
Pre-Relative 1 RM	20 kg CMJ pre/post % change	−0.526	0.044
Pre-Relative 1 RM	0 kg SJ peak power pre/post % change	−0.586	0.022
Pre-Relative 1 RM	20 kg SJ peak power pre/post % change	−0.589	0.021
Pre-Relative 1 RM	Absolute 1RM T1-T2 % change	−0.544	0.036
Pre-Relative 1 RM	Relative 1RM T1-T2 % change	−0.517	0.048

**Table 8:** Vertical Jump% change after 11 weeks of training.

Subjects	0 kg SJ	Effect Size	0 kg CMJ	Effect Size	20 kg SJ	Effect Size	20 kg CMJ	Effect Size
All subjects *n* = 16	14.7% *	0.839 (moderate)	15.8% *	1.379 (large)	18.1% *	0.834 (moderate)	16.6% *	1.102 (moderate)
Strong *n* = 4	7.6%	0.553 (small)	9.9%	0.598 (moderate)	4.8%	0.325 (small)	9.6%	0.532 (small)
Moderate *n* = 4	0.3%	0.013 (trivial)	11.0%	0.583 (moderate)	8.4%	0.406 (small)	9.0%	0.435 (small)
Weak *n* = 7	25.6% *	1.403 (large)	23.8% *	1.377 (large)	28.2% *	1.305 (large)	27.9% *	1.447 (large)

* indicates significance (*p <* 0.05).

**Table 9:** Vertical Jump between group effect size after 11 weeks of training.

Groups	0 kg SJ	0 kg CMJ	20 kg SJ	20 kg CMJ
Moderate–Strong	−0.360 (Small)	0.244 (Small)	−0.589 (Small)	−0.304 (Small)
Moderate–Weak	0.197 (Trivial)	0.337 (Small)	0.270 (Small)	0.387 (Small)
Strong–Weak	0.609 (Moderate)	0.612 (Moderate)	0.940 (Moderate) *	0.730 (Moderate) *

* indicates significance (*p <* 0.05).

**Table 10:** Pre-Post percent change in vertical jump peak power.

Group	0 kg SJ	20 kg SJ	0 kg CMJ	20 kg CMJ
Strong	4.36%	7.66%	4.51%	4.96%
Moderate	4.42%	3.29%	8.52%	4.94%
Weak	20.51%	18.72%	12.56%	11.68%

**Table 11:** Dynamic strength% change after 11 weeks of training.

Group	Back Squat% Change	Effect Size	Relative Back Squat% Change	Effect Size
Strong	11.3%	1.385 (Large)	10.4%	1.589 (Large)
Moderate	18.2%	1.871 (Large)	14.0%	1.587 (Large)
Weak	25.9%	2.361 (Very Large)	23.1%	2.789 (Very Large)

**Table 12:** Between group effect size for dynamic strength.

Group	Back Squat	Relative Back Squat
Moderate–Strong	−0.318 (Small)	−1.373 (Large)
Moderate–Weak	0.769 (Moderate)	0.783 (Moderate)
Strong–Weak	1.129 (Large)	2.322 (Very Large)
